# The Relationship Between Health Literacy, Social Support, Depression, and Frailty Among Community-Dwelling Older Patients With Hypertension and Diabetes in China

**DOI:** 10.3389/fpubh.2020.00280

**Published:** 2020-06-30

**Authors:** Yan Liu, Hongdao Meng, Naidan Tu, Danping Liu

**Affiliations:** ^1^Department of Health Related Social and Behavioral Science, West China School of Public Health and West China Fourth Hospital, Chengdu, China; ^2^School of Aging Studies, College of Behavioral and Community Sciences, University of South Florida, Tampa, FL, United States; ^3^Department of Psychology, College of Arts and Sciences, University of South Florida, Tampa, FL, United States

**Keywords:** frailty, social support, depression, health literacy, older, hypertension, diabetes, China

## Abstract

Population aging is one of the major challenges facing modern society and has attracted global attention. With population aging becoming a global phenomenon, the impact of age-related diseases on health is increasing rapidly. Frailty is one of the most pressing issues facing older adults. The purpose of this study was to explore the interrelationship between health literacy, social support, depression, and frailty among older patients with hypertension and diabetes in China. No studies have investigated the mediating effects of social support and depression between health literacy and frailty. The findings of this study can be applied to help ameliorate frailty in older hypertensive and diabetic patients. Data were collected from 637 older hypertensive and diabetic patients aged 65 years and older in Sichuan Province, China. We used structural equation modeling (SEM) to test the hypothesized relationship among the variables. The results showed that 42.4% of the participants suffered from frailty. The mean scores for health literacy, social support, depression, and frailty were 13.6 ± 5.7, 35.7 ± 6.5, 4.0 ± 3.4, and 3.5 ± 2.1, respectively. Social support had a direct negative association with frailty (β = −0.128, 95%CI: [−0.198, −0.056]), and depression had a direct positive association with frailty (β = 0.326, 95%CI: [0.229, 0.411]), while social support had no direct association with depression. Health literacy had a direct positive association with social support (β = 0.151, 95%CI: [0.077, 0.224]) and a direct negative association with depression (β = −0.173, 95%CI: [−0.246, −0.1]), while health literacy had an indirect negative association with frailty through the mediating effect of social support and depression. To mitigate frailty in older patients with hypertension and diabetes, measures that provide social support, and enhance health literacy, while alleviating depression, should be considered, along with greater attention to patients who are divorced, widowed, or unmarried, those with comorbidities, and those with lower socioeconomic status.

## Introduction

Population aging is a major challenge facing modern society and has attracted increasing global attention. It is estimated that by 2050, one in six people will be over 65 (16%), and the number of people aged 80 and over is expected to triple from 143 million in 2019 to 426 million (1–3). China has the largest older population and has become one of the fastest aging countries in the world (4). According to current projections, China's population will peak in 2028, with a shrinking labor force and an over-65 population of 240 million.

Frailty is one of the most pressing challenges facing older adults as they age (5). Frailty is a comprehensive concept that must consider the complex interplay among multiple factors, and can normally be reflected in three aspects: physical, psychological, and social frailty (6). Frailty can increase the risk of adverse health outcomes, including falls, disability, hospitalization, poor quality of life, and eventually mortality (7–10). In China, hypertension and diabetes affect at least 57 and 26.5% of the older population, respectively (11, 12). The prevalence of frailty is 80% in older patients with hypertension, and is 3–5-fold higher in older patients with diabetes compared with that in those without these conditions (13, 14). Diabetes often coexists with hypertension among older adults in China. In addition, previous studies have shown that frail older adults with hypertension and diabetes have worse physical performance, mental status, and prognosis, as well as higher mortality, compared to non-frail older adults with hypertension and diabetes (15, 16). The above findings emphasize the importance and need for concern about frailty in older patients with hypertension and diabetes within the context of a rapidly aging population.

Health literacy is defined by the WHO as a cognitive and social skill that determines an individual's motivation and ability to access, understand, and use information in a way that promotes and maintains health (17). The results of China's first survey on health literacy showed that older adults had a low health literacy level, with only 3.81% of people over 65 years of age showing adequate health literacy skills (18). Low health literacy has been reported to be associated with a poor understanding of one's medical condition, poor self-care, delayed care-seeking when symptomatic, and lower use of preventive services, and can also affect disease management and outcomes in chronically ill patients (19, 20). Adequate health literacy can prevent frailty in older adults and plays a positive role in the management and intervention of community-dwelling frail older population (21). Health literacy is also associated with social support, and low health literacy limits an individual's ability to access resources from social networks (22).

Social support is a multidimensional concept, usually defined as “perceived and received social resources or help.” These can be instrumental, emotional, or informational (23). As a developing country, China is experiencing rapid urbanization and economic development (24), which has led to changes in the cultural norms of filial piety in eldercare as well as in traditional family size (25). The geographical distance between older adults and their children and the proportion of female participation in full-time employment are increasing. In addition, since the implementation of the “One Child Policy” from 1979, the rate of population aging has increased and traditional Chinese large-sized households have gradually been replaced by the nuclear family (24). All these factors have made it increasingly difficult for family members to provide care and support to older relatives. Woo et al. found that increasing social support among older adults was associated with reduced frailty (26). Strong empirical evidence suggests that people with better social support are less likely to suffer from physical and mental illness and that sufficient social support can help alleviate the frailty condition of community-dwelling older people (27, 28). In addition, Patra et al. argued that social support can effectively reduce patients' psychological distress, such as depression (29). Social support was found to be a significant predictor of depression among older hypertensive patients and adults with diabetes (30, 31). Furthermore, Stewart et al. demonstrated that social support could be a mediator of the relationship between health literacy and depression among smokers (32).

Globally, depression is one of the most common psychiatric disorders among people aged 65 and above, which affects one in seven older adults (29). It has been suggested that depression and frailty often coexist, and the prevalence of depression is as high as 46.5% among older people with frailty (33). Depression was proven to be a strong predictor of frailty in a study of women with disabilities (34). A prospective cohort study demonstrated that depressive symptoms were associated with an increased risk of becoming frail after adjusting for antidepressant use and other important covariates in women 65 and older (35). In addition, evidence has linked higher depression levels with limited or poor health literacy in older adults, and depression is found to mediate the relationship between health literacy and health outcomes among breast cancer patients (36, 37).

Most previous studies in this area have investigated the associations between social support, health literacy, depression, and frailty, but the combined effects of these factors on frailty and the underlying mechanisms of these relationships remain unclear. In addition, few studies have examined the frailty status of a particular group—older patients with hypertension and diabetes. Therefore, the aim of the present study was to examine the frailty status and the interrelationships, as well as potential underlying mechanisms, between social support, health literacy, depression, and frailty among community-dwelling older individuals with hypertension and diabetes living in China.

**Figure 1:**
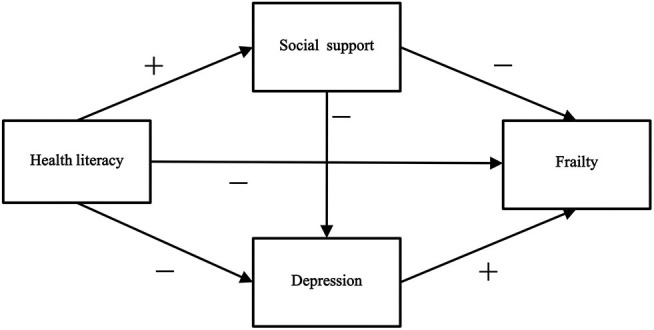
The theoretical model and hypotheses.

A structural equation model was constructed to explore the relationship between health literacy, social support, depression, and frailty among older hypertensive and diabetic patients. Based on previous research, we proposed 10 hypotheses (shown in Table 1) and a theoretical model (Figure 1). The findings of this study identified the key factors that contribute to the prevention and control of frailty in older patients, which is of great significance for decreasing frailty and improving the health of older patients with hypertension and diabetes.

**Table 1:** The theoretical hypotheses.

**Hypotheses**
1. Health literacy has a direct positive effect on social support.
2. Health literacy has a direct negative effect on depression.
3. Health literacy has a direct negative effect on frailty.
4. Social support has a direct negative effect on frailty.
5. Social support has a direct negative effect on depression.
6. Depression has a direct positive effect on frailty.
7. The relationship between health literacy and depression is mediated by social support.
8. The relationship between social support and frailty is mediated by depression.
9. The relationship between health literacy and frailty is mediated by social support.
10. The relationship between health literacy and frailty is mediated by depression.

## Materials and Methods

### Ethical Considerations

The study protocol was approved by the Ethics Committee of Sichuan University. The survey was voluntary, and informed consent was obtained from each participant before the interview was held. The participants were informed that their anonymity will be guaranteed.

### Participants and Procedure

This cross-sectional study was conducted between December 2017 and May 2018 in Sichuan Province, China. All community-dwelling elders aged 65 years and older who had been diagnosed with hypertension and diabetes were eligible to participate.

A multistage stratified random sampling process was used to acquire the sample. In the first stage, we randomly selected a city in Sichuan Province. In the second stage, 10 communities of the city were randomly selected as survey areas. In the third stage, systematic sampling was used to obtain 70 subjects from the database of hypertensive and diabetic older patients established by each community hospital. A total of 700 patients were surveyed face-to-face by professionally trained investigators. To ensure the quality of the investigation, we set the following exclusion criteria: (a) patients who could not adequately answer questions due to cognitive impairment; (b) those unable to cooperate for personal reasons (e.g., migration, rejection). Ultimately, we obtained and analyzed 637 (91%) valid responses.

### Measures

Sociodemographic data and information on health literacy, social support, depression, and frailty were collected using the questionnaires.

#### Sociodemographic Characteristics

The sociodemographic characteristics assessed included gender, age, marital status, education level, individual annual income, per capita annual household income, smoking status, drinking status, and comorbidities. Age was categorized as 65 – 69, 70 – 74, 75 – 79, and ≥80 years. Marital status was defined as a binary variable: “Married” or “Divorced, widowed, or unmarried.” Education level was classified as “No formal education,” “Primary school” “Middle school,” or “High school and above.” Individual annual income and per capita annual household income were both divided into five categories: < $750, $750 – 1,499, $1,500 – 4,499, $4,500 – 7,499, and ≥$7,500. Current smoking status, current drinking status, and presence of comorbidities were binary variables (“Yes” or “No”).

#### Health literacy

The health literacy scale was constructed by referring to the Chinese Citizen Health Literacy Questionnaire developed by the National Health Commission of the People's Republic of China (38). The expert consultation method was used to select representative questions that are closely related to health literacy for older adults. To improve the questionnaire, we added further questions assessing knowledge of common chronic disease prevention. The full scale contained three dimensions: knowledge and belief literacy, behavior literacy, and skill literacy, with 33 items and a total score of 33. The respondents who correctly answered 80% or more of the questions were regarded as having good health literacy. In the current study, Cronbach's α of the scale was 0.832.

#### Social Support

Social support was measured using the Social Support Rating Scale (SSRS) (39) designed by Xiao et al. This scale consists of 10 items and contains three subscales: objective support, subjective support, and support utilization. Responses are given on a 4-point Likert scale, and the total score can range from 12 to 66, with higher scores indicating stronger social support. Objective support includes three items, with a total score ranging from 1 to 22; subjective support includes four items, with a total score ranging from 8 to 32; and support utilization includes 3 items, with a total score ranging from 3 to 12. The total score is divided into three levels: low (12–22), moderate (23–44), and high (45–66). The SSRS has demonstrated good to excellent reliability in prior studies, with a Cronbach's α of 0.89 and a test–retest reliability of 0.92 (39). In the current study, Cronbach's α of the scale was 0.735.

#### Depression

The short version of the Center for Epidemiologic Studies Depression Scale (CES-D-10) was used to measure depressive symptoms during the survey; this scale consists of 10 items from the original 20-item CES-D developed by Anderson et al. (40). The CES-D-10 includes depressive affect (three items), somatic symptoms (five items), and positive affect (two items) (41). Each of the 10 items is rated on a 4-point Likert scale, ranging from “rarely (<1 day) = 0” to “most or all of the time (5–7 days) = 3,” and the summed scores of the items can range from 0 to 30. Higher scores represent greater levels of symptom frequency, and a score of 10 or higher indicates significant depressive symptoms (40). The CESD-10 has shown excellent reliability (Cronbach's α of 0.70 and composite reliability of 0.72) (42). In the current study, Cronbach's α of the scale was 0.714.

#### Frailty

Frailty was assessed by the Chinese version of the Tilburg Frailty Indicator Scale (TFI) designed by Dong et al. (6), which is a standardized self-reported questionnaire containing 15 items addressing three domains: physical, psychological, and social. The physical domain (0–8 points) consists of eight items related to physical health, unexplained weight loss, difficulty walking, balance, hearing problems, vision problems, strength in hands, and physical tiredness. The psychological domain (0–4 points) comprises four items corresponding to cognition, depressive symptoms, anxiety, and coping. The social domain (0–3 points) includes three items related to living alone, social relations, and social support. The scale items are scored using a binary scoring method that ranges from 0 to 15 points; the higher the score, the higher the patient's frailty. Two items of the scale have three response categories (yes, no, and sometimes). “Yes” or “sometimes” responses are scored one point each, while “no” responses are scored zero. The cutoff for frailty in the Chinese version of the TFI is 4. The Chinese TFI has good validity and reliability (Cronbach's α = 0.71) as an integral instrument to measure the frailty of Chinese community-dwelling elders (6). In the current study, Cronbach's α of the scale was 0.597.

### Statistics Analysis

The data were entered using the Epidata3.1 database. Statistical analyses were conducted using IBM SPSS Statistics 20.0 (IBM Corporation, Armonk, NY, USA) and AMOS 24.0 (IBM, New York, NY, USA). First, we used descriptive statistics to examine participant characteristics. Proportions and frequencies were calculated for the sociodemographic characteristics, and means and standard deviations were calculated for the study variables. Second, Spearman's correlation coefficient was used to analyze the correlations between health literacy, social support, depression, and frailty. Third, a linear regression model was also used to analyze the influence of sociodemographic factors, in addition to health literacy, social support, and depression on frailty status. Fourth, a structural equation model (SEM) was constructed to further test hypothesized relationships among social support, health literacy, depression, and frailty of older patients with hypertension and diabetes.

The SEM used bootstrap maximum likelihood estimation. To evaluate the overall model fit, we applied the following model fit criteria: an adjusted goodness of fit index (AGFI), goodness of fit index (GFI), comparative fit index (CFI), and Tucker–Lewis index (TLI) of 0.90 or above; a standardized root mean square residual (SRMR) and a root mean squared error of approximation (RMSEA) <0.08; and an χ^2^/df of <3. These criteria indicated an acceptable model fit. Statistical significance was set at *p* < 0.05.

## Results

### Sociodemographic Characteristics of the Participants

The sociodemographic characteristics of the 637 elder patients with hypertension and diabetes are presented in Table 2. In the sample, there were more females (64.8%) than males (35.2%). The average age of the participants was 72.21 years (*SD* = 5.76), and the highest proportion was people aged 65–69 years (38.8%). Most were married (75%), had an education of primary school or less (81.2%), had an individual annual income of $1,500–4,499 (58.6%), and did not currently smoke (87.1%), or drink (85.4%). Overall, 35.3% of the participants had a per capita annual household income of $1,500–4,499, and 25% had chronic diseases other than hypertension or diabetes.

**Table 2:** Sociodemographic characteristics of the participants (*n* = 637).

**Sociodemographic characteristics**	***n***	**%**
**Gender**		
Female	413	64.8
Male	224	35.2
**Age, Group**		
65–69	247	38.8
70–74	193	30.3
75–79	118	18.5
≥80	79	12.4
**Marital Status**		
Married	478	75.0
Divorced, widowed, or unmarried	159	25.0
**Education Level**		
No formal education	207	32.5
Primary school	310	48.7
Middle school	77	12.1
High school and above	43	6.8
**Individual Annual Income ($)**		
<750	106	16.6
750–1,499	60	9.4
1,500–4,499	373	58.6
4,500–7,499	82	12.9
≥7,500	16	2.5
**Per Capita Annual Household Income ($)**		
<750	57	8.9
750–1,499	54	8.5
1,500–4,499	225	35.3
4,500–7,499	200	31.4
≥7,500	101	15.9
**Current Smoking Status**		
No	555	87.1
Yes	82	12.9
**Current Drinking Status**		
No	544	85.4
Yes	93	14.6
**Comorbidities**		
No	478	75.0
Yes	159	25.0

### Descriptive Analysis of Study Variables

The score of each variable is shown in Table 3. The mean scores for health literacy, social support, depression, and frailty were 13.6 ± 5.7, 35.7 ± 6.5, 4.0 ± 3.4, and 3.5 ± 2.1, respectively. The proportion of individuals who had good health literacy was only 3.9% (25). Based on the score, 2.2% (14), 87.8% (559), and 10% (64) of older hypertensive and diabetic patients experienced low, moderate, and high social support, respectively. In addition, 8.3% (53) had depressive symptoms, and 42.4% (270) suffered from frailty.

**Table 3:** Description of social support, health literacy, depression, and frailty scores (*n* = 637).

**Contents**	**Range**	**Mean (*SD*)**
**Health literacy**	0–33	13.6 ± 5.7
Knowledge and belief literacy	0–22	7.9 ± 4.3
Behavior literacy	0–9	4.5 ± 1.7
Skill literacy	0–2	1.2 ± 0.5
**Social support**	12–66	35.7 ± 6.5
Objective support	1–22	7.5 ± 2.2
Subjective support	8–32	21.5 ± 4.1
Support utilization	3–12	6.7 ± 2.0
**Depression**	0–30	4.0 ± 3.4
**Frailty**	0–15	3.5 ± 2.1

### Correlations of the Study Variable

Spearman's correlations for the study variables are presented in Table 4. Health literacy had a positive correlation with social support and a negative correlation with depression, while social support had a negative correlation with frailty. Depression was significantly positively correlated with frailty.

**Table 4:** Correlation coefficients among study variables.

**Variable**	**(1)**	**(2)**	**(3)**	**(4)**
(1) Health literacy				
(2) Social support	0.168^**^			
(3) Depression	−0.166^**^	−0.003		
(4) Frailty	−0.061	−0.102^**^	0.180^**^	

** *p < 0.01*.

### Linear Regression Analysis

We used frailty as the dependent variable and sociodemographic variables, social support, health literacy, and depression as independent variables in a multiple linear regression model. Table 5 presents the statistically significant variables in the analysis (*p* < 0.05). The results showed that in addition to health literacy, social support, and depression, eight sociodemographic factors were influencing factors of frailty in hypertensive and diabetic elders. These included age, marital status, educational level, individual annual income, per capita annual household income, current smoking status, current drinking status, and presence of comorbidities.

**Table 5:** Linear regression of factors associated with frailty.

**Factor**	**Unstandardized coefficients**		**Standardized coefficients**	***t***	***p*-Value**
	***B***	***SE***	**β**		
Constant	4.267	0.563		7.961	<0.001
**Gender (ref: female)**					
Male	−0.351	0.193	−0.080	−1.819	0.069
**Age (ref: 65–69)**					
70–74	0.37	0.184	0.081	2.009	0.045
75–79	0.101	0.215	0.019	0.473	0.636
≥80	0.471	0.257	0.074	1.83	0.068
**Marital status (ref: married)**					
Divorced, widowed, or unmarried	0.402	0.189	0.083	2.126	0.034
**Education level (ref: no formal education)**					
Primary school	−0.529	0.183	−0.127	−2.889	0.004
Junior middle school	−0.916	0.262	−0.143	−3.493	0.001
High school and above	−0.428	0.374	−0.051	−1.145	0.253
**Individual annual income (ref:** **<750, $)**					
750–1,499	−0.724	0.353	−0.101	−2.055	0.04
1,500–4,499	−0.63	0.277	−0.148	−2.275	0.023
4,500–7,499	−0.929	0.353	−0.149	−2.635	0.009
≥7,500	−1.745	0.582	−0.131	−2.999	0.003
**Per capita annual household income (ref: <750, $)**					
750–1,499	−0.898	0.41	−0.12	−2.192	0.029
1,500–4,499	0.273	0.36	0.062	0.76	0.448
4,500–7,499	0.17	0.352	0.038	0.481	0.631
≥7,500	0.618	0.396	0.108	1.561	0.119
**Current smoking status (ref: NO)**					
YES	0.834	0.259	0.133	3.218	0.001
**Current drinking status (ref: NO)**					
YES	−0.556	0.237	−0.094	−2.346	0.019
**Comorbidities (ref: NO)**					
YES	0.446	0.18	0.092	2.481	0.013
Health literacy	0.026	0.016	0.07	1.659	0.098
Social support	−0.025	0.013	−0.079	−2.034	0.042
Depression	0.179	0.023	0.292	7.792	<0.001

*R^2^ = 0.237, F = 8.655, p <0.001*.

The frailty score of patients aged 70–74 years (*p* = 0.045) was on average 0.37 points higher than that of patients aged 65–69 years. The frailty score of divorced, widowed, or unmarried participants (*p* = 0.034) was on average 0.402 points higher than that of married participants. The frailty scores of patients who were educated to primary school level (*p* = 0.004) and those of patients who were educated to middle school level (*p* = 0.001) were on average 0.529 and 0.916 points lower than the frailty scores of patients who were educated to less than primary school level. For individual annual income, the frailty scores of patients in the 750–$1,499 (*p* = 0.004), 1,500–$4,499 (*p* = 0.023), 4,500–$7,499 (*p* = 0.009), and ≥$7,500 (*p* = 0.003) income groups were on average 0.724, 0.63, 0.929, and 1.745 points lower than that of patients in the < $750 income group, respectively. The frailty score of subjects who had a per capita annual household income of 750–$1,499 (*p* = 0.029) was on average 0.898 points lower than that of subjects who earned < $750. The frailty score of subjects who currently smoked (*p* = 0.001) was on average 0.834 points higher than that of non-smokers, and the frailty score of subjects who currently consumed alcohol (*p* = 0.019) was on average 0.556 points lower than that of those who did not. In addition, the frailty score of older patients with two or more chronic diseases (*p* = 0.013) was on average 0.446 points higher than that of patients with none.

### Test of the Study Model

**Figure 2:**
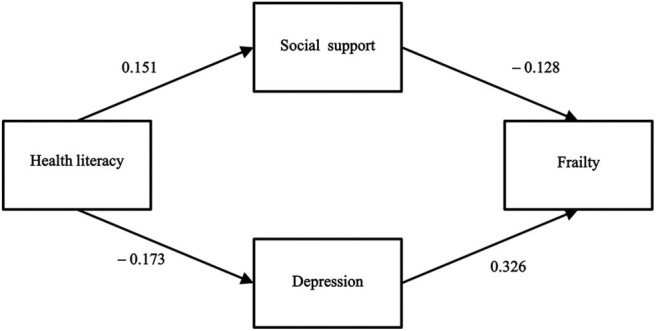
The final model and standardized model paths.

By establishing the SEM, the four research variables were connected with each other, and the data and the established theoretical model were fitted by the maximum likelihood estimation method. The results showed that the link between health literacy and frailty and the link between social support and depression were not statistically significant. Therefore, we modified the model by removing these two paths. With the addition of sociodemographic factors as covariates, the arrow direction among the core variables in the SEM remained unchanged, and the corresponding coefficients did not change significantly. Thus, the sociodemographic factors were not confounding factors. Figure 2 shows the final model and the correlation and effect paths among the key variables. The goodness of fit tests showed AGFI, GFI, CFI, TLI, SRMR, RMSEA, and χ^2^/df values of 0.992, 0.998, 1.0, 1.0, 0.0179, 0.011, and 1.075, respectively, indicating that the model fit the data well.

Table 6 presents the test results of the hypothesis model, and each path adopted bias-corrected bootstrapping with 2,000 replications using maximum likelihood estimation. As expected, health literacy had a direct positive association with social support (β = 0.151, 95%CI: [0.077, 0.224]) and a direct negative association with depression (β = −0.173, 95%CI: [−0.246, −0.1]). These two results support Hypotheses 1 and 2. However, health literacy had only an indirect negative association with frailty (β = −0.076, 95%CI: [−0.113, −0.046]), rather than a direct association, leading us to reject Hypothesis 3. Social support had a direct negative association with frailty (β = −0.128, 95%CI: [−0.198, −0.056]), thus supporting Hypothesis 4. Depression had a direct positive association with frailty (β = 0.326, 95%CI: [0.229, 0.411]). Hypothesis 6 was also supported. The results showed that social support had no direct association with depression, leading us to reject Hypotheses 5, 7, and 8.

**Table 6:** Direct, indirect, and total effects and 95% confidence intervals for the final model.

**Model pathways**	**Estimated effect**	**95%CI**
**Total effects**		
Social support < –Health literacy	0.151	[0.077, 0.224]
Depression < –Health literacy	−0.173	[−0.246, −0.1]
Frailty < –Health literacy	−0.076	[−0.113, −0.046]
Frailty < –Social support	−0.128	[−0.198, −0.056]
Frailty < –Depression	0.326	[0.229, 0.411]
**Direct effects**		
Social support < –Health literacy	0.151	[0.077, 0.224]
Depression < –Health literacy	−0.173	[−0.246, −0.1]
Frailty < –Social support	−0.128	[−0.198, −0.056]
Frailty < –Depression	0.326	[0.229, 0.411]
**Indirect effects**		
Frailty < –Health literacy	−0.076	[−0.113, −0.046]

Table 6 shows that the total effect and indirect effect between health literacy and frailty are statistically significant, which means that mediating effects exist. Table 7 presents the results of significance testing of the mediating pathways. If the 95% confidence interval does not include zero, the mediating effect is statistically significant. The results illustrated that the relationship between health literacy and frailty was mediated by social support (95%CI: [−0.013, −0.002]) and depression [−0.031, −0.011], thus supporting Hypotheses 9 and 10. Based on the above, social support and depression could directly affect frailty, and health literacy could indirectly affect frailty through social support and depression.

**Table 7:** Significance tests of mediating pathways.

**Model pathways**	**95%CI**
Frailty < –Social support < –Health literacy	[−0.013, −0.002]
Frailty < –Depression < –Health literacy	[−0.031, −0.011]

## Discussion

This study aimed to clarify the interrelationships between health literacy, social support, depression, and frailty among older patients with hypertension and diabetes in China. To the best of our knowledge, this is the first study reporting the mediating roles of social support and depression between health literacy and frailty in older patients with hypertension and diabetes. The findings here can provide possible clues and propose directions for the development and implementation of intervention strategies and measures to ameliorate the frailty of older hypertensive and diabetic patients.

Overall, we found that 42.4% of older hypertensive and diabetic patients suffered from frailty, which is higher than that reported for studies on the older population in Padua, Italy (21.9%) (43), and Northern Germany (41.4%) (44). The higher prevalence of frailty found in this study is understandable. Physiological function and the ability of the body to respond to various external stresses declines progressively with age, making frailty more likely to occur in older individuals (2, 4). Additionally, hypertension and diabetes can increase vulnerability by compromising the metabolic balance and cardiovascular performance (1). Moreover, 25% of the patients in this study had multiple comorbidities; these chronic diseases and conditions can collectively accelerate frailty through distinct mechanisms (45, 46).

This study found that only 10% of older hypertensive and diabetic patients have high social support. Of the three dimensions of social support, the objective support and the support utilization were relatively poor, achieving mean scores of 7.5 ± 2.2 and 6.7 ± 2.0, respectively, which is similar to a previous study (47). As the cultural norms of filial piety in eldercare have changed, the younger generations are increasingly likely to provide financial support to compensate for their inability to offer physical support or companionship to their parents (24). Meanwhile, social changes and demographic changes are eroding traditional family support networks by altering the family structure and the size of the older population, many of whom have been long separated from their children and do not have other avenues of emotional support (48). In addition, as people age, their physical activity is restricted and social networks further shrink, leading to few sources of social support in daily life except for visits from their children (47).

As predicted, older hypertensive and diabetic patients who have stronger social support were found to be less subject to frailty, consistent with existing studies (26). Berglund et al. (49) and Strawbridge et al. (50) state that satisfying the increasing physical and psychological requirements of older people can improve their life satisfaction and may help to alleviate frailty. One possible explanation for this may be that better social security and welfare, facilitated medical and sanitary conditions, and more resources and social interactions can be offered by effective social support. Moreover, older people can develop the necessary social companionship, instrumental aid, and emotional comfort from social networks to alleviate their negative feelings and more effectively nurture their ability to deal with disease (51, 52). Therefore, providing sufficient social support represents an effective strategy to mitigate frailty in older hypertensive and diabetic patients.

The results of this study showed that in the 637 enrolled patients, 8.3% developed significant depressive symptoms, which is significantly higher than that of the general population (0.56%) (53). The increase in the aging population is expected to give rise to a range of challenges. First, social security and financial support for older people have become relatively limited. Second, being separated from their children or partners can cause older people to become depressed and isolated and to lack emotional comfort (54, 55). Finally, may problems can occur with aging, [e.g., cognitive decline, sleep disturbances, and malnutrition (56)]. Previous studies have reported that hypertension and diabetes are characterized by a long course, a poor prognosis, and are difficult to cure. These conditions continuously reduce a person's quality of life, leading to emotional disorders, a psychological burden, and reduced happiness, thereby causing depression over time (57, 58). Thus, it is expected that older hypertensive and diabetic adults experience a relatively high level of depression.

Our findings support the existence of a specific association between depression and frailty (59). This finding in line with previous studies showing that depression is linked to the onset of frailty and that people with depression are more likely to experience frailty (15, 60). Depression commonly manifests as cognitive impairment, with insufficient coping resources in psychological and social areas, and older adults can lose interest in physical and social activities, elevating the risk of decreased physical function and falls. As indicated by a meta-analysis, depression and frailty may share common risk factors and pathophysiological pathways, and patients subject to depressive disorder often exhibit frailty at significantly high levels (33), revealing that depression may be a vital target to mitigate and prevent frailty and its consequences. Previous studies have indicated that social support is a significant predictor of depression (30, 31). In this study, however, poor social support was not identified to be associated with higher levels of depressive symptoms, which is in agreement with Ang et al. (61). These discrepancies require further interpretation and reflection.

Consistent with existing findings, the participants showed rather low health literacy, with a mean score of 13.6 ± 5.7. Only 3.9% of the older hypertensive and diabetic patients achieved good health literacy, suggesting that low health literacy was a widespread phenomenon (62, 63). Health literacy can depend on age, education level, and economic status (64). In China, the health literacy level was shown to decline with age in older adults (65). Participants with a lower education level were inclined to have lower health literacy, probably due to their limited ability to acquire health information and poor acceptance of knowledge (65). In our sample, 81.2% of participants had a primary school level of education or below. Participants with lower income levels achieved lower levels of health literacy for their lower social status and material level, and they showed low use of health education resources (66). In the present study, the proportion of the participants with individual annual incomes and per capita annual household incomes below $4,500 were 84.6 and 52.7%, respectively.

The findings here support the hypothesis that health literacy acts as a direct predictive factor for both social support and depression, consistent with previous studies (22, 32). Earlier studies reported that individuals with lower health literacy often hide their health problems from family, friends, and healthcare providers because of shame, thereby causing them to likely experience isolation and to perceive less available support (67). Moreover, another study indicated that health literacy can act as an asset and empowerment tool that helps individuals access education about mental health services and utilize existing mental health services (68). Accordingly, to decrease depression and boost social support among older hypertensive and diabetic patients, efforts should be made by health care providers and policymakers to improve the health literacy of these patients.

This study also investigated whether higher levels of health literacy are negatively correlated with frailty. In contrast to our predictions, the model fitting the conceptual model was acceptable. Health literacy was not a direct predictor of frailty, whereas it could impact frailty by intermediating social support and depression. This finding is in accordance with the results of Shah et al. who studied male veterans (69). The lack of association of health literacy found in this study compared with existing studies may have arisen owing to differences in the characteristics of the participants.

We also identified that older hypertensive and diabetic patients who were divorced, widowed, or unmarried or those with two or more chronic diseases were more likely to experience frailty, which is consistent with existing studies (15, 70). Additionally, six other sociodemographic characteristics of the older hypertensive and diabetic patients, namely, age, education, personal and family incomes, drinking status, and smoking status, showed associations with frailty. Education and income were the two socioeconomic status (SES) factors that were most strongly inversely associated with frailty. Poli et al. (71) highlighted that older subjects with higher SES had a significantly lower risk of being frail, indicating that practical ways to assist older hypertensive and diabetic patients subject to lower SES may help relieve their frailty.

A strength of this study was that we focused on a rapidly growing vulnerable population—older patients subject to hypertension and diabetes—and the findings here stressed the mediating roles of social support and depression between health literacy and frailty. This study also had some limitations. First, the participants were representative of patients that had hypertension and diabetes in the Sichuan province of China and the findings might differ according to population or geographic region. Second, the cross-sectional and self-reporting nature of the study may have introduced measurement and/or recall bias. Some selection bias also existed as the participants were ≥65 years old. In addition, to draw further inferences from the data, a more extensive and explanatory longitudinal study is required.

## Conclusions

This study used structural equation modeling to clarify the intermediary paths between health literacy and frailty among community-dwelling elder hypertensive and diabetic patients in China. The results suggest that although health literacy is not a direct predictor of frailty, it could impact frailty by intermediating social support and depression. Thus, far, no studies have investigated these two mediating effects, and this study may help to elucidate the pathogenesis of frailty. The critical sociodemographic variables that indicated higher levels of frailty in older hypertensive and diabetic patients included being divorced, widowed, or unmarried, the presence of comorbidities, and lower levels of education and income. To mitigate frailty in elderly patients subject to hypertension and diabetes, measures that could act effectively to improve social support, depression, and health literacy should be considered. Good health literacy can support the possibility of strong social support and reduced depression, resulting in a low level of frailty. Furthermore, a greater emphasis should be placed on elder hypertensive and diabetic patients who are divorced, widowed, or unmarried, those with comorbidities, and those with lower SES.

## Data Availability Statement

The datasets presented in this study can be found in online repositories. The names of the repository/repositories and accession number(s) can be found in the article/supplementary material.

## Author Contributions

DL conceived the study and prepared the study protocol. YL performed the data analysis and drafted the first manuscript. All authors contributed to content revisions, approved the final version, and agreed to be responsible for all aspects of the current work.

## Conflict of Interest

The authors declare that the research was conducted in the absence of any commercial or financial relationships that could be construed as a potential conflict of interest.
